# Supplementary material for: The genome sequence of a snail-killing fly, Dichetophora obliterata (Fabricius, 1805)
Source: Wellcome Open Res. 2025 Apr 8;10:176. [Version 1] doi: 10.12688/wellcomeopenres.23993.1 (PMC12107236; doi:10.12688/wellcomeopenres.23993.1)
Supplement: Peer review history

# Reviewer report: Reviewer response for version 1

**DOI:** 10.21956/wellcomeopenres.26470.r122489

**Published:** 2025-05-26

**Authors:** Taro Nakamura

**Affiliations:** 1 National Institute for Basic Biology, Okazaki, Japan

This manuscript reports the genome sequencing of *Dichetophora obliterata*, a parasitic snail-killing fly. The assembled genome size is approximately 1.3 Gb, and high-quality chromosome-level assembly and annotation have been achieved. The genome analysis is clearly presented, demonstrating high data quality and notable scientific contribution.

 The methodologies used in this study are clearly and comprehensively described, reaching a standard that allows for reproducibility by third parties.

 The application of PacBio HiFi long-read sequencing combined with Hi-C technology has provided a high-quality, chromosome-level genome assembly. Quality metrics such as the QV scores and BUSCO analysis further support the high quality of the generated data.

Are sufficient details of methods and materials provided to allow replication by others?

Yes

Is the rationale for creating the dataset(s) clearly described?

Yes

Are the datasets clearly presented in a useable and accessible format?

Yes

Are the protocols appropriate and is the work technically sound?

Yes

Reviewer Expertise:

Developmental Biology

I confirm that I have read this submission and believe that I have an appropriate level of expertise to confirm that it is of an acceptable scientific standard.

# Reviewer report: Reviewer response for version 1

**DOI:** 10.21956/wellcomeopenres.26470.r122172

**Published:** 2025-05-08

**Authors:** Denise Gemmellaro

**Affiliations:** 1 Department of Biological Sciences, Kean University, Union, New Jersey, USA

The authors assembled the complete genome of a female specimen of *Dichetophora obliterata. *This is a dipteran belonging to the family Sciomyzidae. The genome sequence is 1,312.79 megabase long and 15,139 protein coding genes were identified. 

 This is a very straight forward paper presenting a clear, complete and reproducible protocol for genome analysis, from DNA extraction to bioinformatics.

Are sufficient details of methods and materials provided to allow replication by others?

Yes

Is the rationale for creating the dataset(s) clearly described?

Yes

Are the datasets clearly presented in a useable and accessible format?

Yes

Are the protocols appropriate and is the work technically sound?

Yes

Reviewer Expertise:

Entomology, Forensic Entomology, Diptera, Decomposition Ecology

I confirm that I have read this submission and believe that I have an appropriate level of expertise to confirm that it is of an acceptable scientific standard.
